# The "Rewa" Outrage

**Published:** 1918-01-19

**Authors:** 


### THE "REWA" OUTRAGE.

THE time has long since passed when even the most credulous and charitable person can believe that any words of remonstrance or sense of shame will exercise a restraining influence on the actions of the German Government. We are in the region of hard facts, and these plainly show that if the Germans can inflict harm on their enemies they will not hesitate at any conduct, however base, ignoble, and cowardly this may be. War, as they understand it, knows neither honour nor pity nor chivalry. The revelation is open to the whole world, and it must rule both opinion and practice. The latest display of this set purpose is the sinking of the hospital ship *Rewa* in the Bristol Channel, though there are precedents enough and to spare. But a few short years ago no one would have credited the suggestion that a civilised nation would have stooped to strike at wounded and helpless soldiers, or that hospitals and hospital ships would be deliberately attacked by bombs or torpedoes. Such an opinion has now disappeared under the pressure of experience. There *is* a nation which does these things and rejoices to do them, and with this state of affairs the peoples of the Allies have to reckon. To indulge in protests or to talk about the claims of humanity is a vain thing, for neither the one nor the other will touch the authors of crimes which are perpetrated as part and parcel of a policy which has for its motive victory by terror. The policy will not succeed, but its adoption and prosecution are records which must surely not be forgotten in the day of reckoning. Our people are neither a hard-hearted nor a revengeful people, but they have a passionate love of justice and of fair play and fair fighting, and no smooth words will convince them that crime should escape punishment. The sinking of the *Rewa* and kindred outrages are in the national memory, and their effects, not in words but in acts, will appear in due season.

There is need, however, to present the position with accuracy. But recently a well-known author advocated in the press a wide dissemination by leaflet of the story of the indignities and cruelties inflicted on British prisoners of war by German civilians and soldiers, his object being to sustain and heighten the resolve of the nation to bring the system responsible for such things to a final and inglorious end. Unfortunately he spoke of his enterprise as the cultivation of hate, and thus incurred censure from clerical and other critics. " Hate " is an attitude of mind which does not readily lend itself to the temper of the British peoples, who indeed regard it as essentially ignoble and unmanly. Had its advocate urged " righteous anger " or " moral indignation " he would have been on safer ground, and his purpose would have excited general sympathy. For ourselves, we are with him entirely in the wish to keep prominent before our countrymen the realities with which we have to deal. The latest of these merely repeats an earlier tale. Against a ship openly announced as carrying hundreds of wounded men a German hand loosed a torpedo on its deadly mission, and, but for good fortune and good discipline, these men, together with their doctors and nurses, would inevitably have perished. The intention succeeded only in part. But its quality and significance remain. This is the ground of judgment, and to suppose that acts of this order are to escape a full penalty is to deny the value of human justice and to indict the moral government of mankind. To urge that they be remembered is a duty not less to humanity than to patriotism. Either criminals against natural law must be brought to justice or civilisation must abandon her task in despair.

